# Electrospun Polyvinylidene Fluoride Piezoelectric Fiber Glass/Carbon Hybrid Self-Sensing Composites for Structural Health Monitoring

**DOI:** 10.3390/s23083813

**Published:** 2023-04-07

**Authors:** Wei-Han Cheng, Ping-Lun Wu, Hsin-Haou Huang

**Affiliations:** Department of Engineering Science and Ocean Engineering, National Taiwan University, Taipei 10617, Taiwan; r11525002@ntu.edu.tw (W.-H.C.); r08525024@ntu.edu.tw (P.-L.W.)

**Keywords:** electrospun fibers, PVDF, multifunctional composites, smart materials, structural health monitoring

## Abstract

In this study, a polyvinylidene fluoride (PVDF)/graphene nanoplatelet (GNP) micro-nanocomposite membrane was fabricated through electrospinning technology and was employed in the fabrication of a fiber-reinforced polymer composite laminate. Some glass fibers were replaced with carbon fibers to serve as electrodes in the sensing layer, and the PVDF/GNP micro-nanocomposite membrane was embedded in the laminate to confer multifunctional piezoelectric self-sensing ability. The self-sensing composite laminate has both favorable mechanical properties and sensing ability. The effects of different concentrations of modified multiwalled carbon nanotubes (CNTs) and GNPs on the morphology of PVDF fibers and the *β*-phase content of the membrane were investigated. PVDF fibers containing 0.05% GNPs were the most stable and had the highest relative *β*-phase content; these fibers were embedded in glass fiber fabric to prepare the piezoelectric self-sensing composite laminate. To test the laminate’s practical application, four-point bending and low-velocity impact tests were performed. The results revealed that when damage occurred during bending, the piezoelectric response changed, confirming that the piezoelectric self-sensing composite laminate has preliminary sensing performance. The low-velocity impact experiment revealed the effect of impact energy on sensing performance.

## 1. Introduction

The global demand for fiber-reinforced polymers (FRPs) has increased in recent years. In addition to having high stiffness, low weight, and corrosion resistance, such combinations of fibers and matrix materials have high flexibility and can be adjusted for different application needs. FRPs are increasingly used in aerospace and energy-related industries, such as in wing casings and wind turbine blades, to replace metal-based materials for reducing weight as well as providing strength. The mechanical properties of FRPs are mainly related to the technology through which they are processed, and the mechanism through which the composite material is damaged due to process defects is complex. Most commonly, damage to a composite is located below the surface and is almost invisible [1,2]; damage assessment and prediction are thus important when fabricating composite materials.

Structural health monitoring (SHM) is an emerging technology developed from nondestructive monitoring technology (NDT). SHM combines advanced sensor and algorithm technologies to monitor structural health, and it improves reliability and safety, reduces life cycle costs, and aids the design of composite materials [3,4] Piezoelectric materials are widely used in sensors and actuators and for energy harvesting because of the electromechanical coupling characteristics of the piezoelectric effect [5,6,7,8]. Common piezoelectric materials include lead zirconate titanate, barium titanate, and polyvinylidene fluoride (PVDF) [9,10,11]. Regarding the monitoring of FRP health, the early monitoring technology mostly involved attaching sensors to the surface of the material to enable external monitoring [12,13,14]. The disadvantage of this method is that the sensor is easily affected by the environment. The performance is low, the monitoring process is cumbersome, and any processes involving the material must be stopped to allow monitoring equipment to be installed. Therefore, embedded-sensor technology has been developed; in this approach, the sensor is embedded in the material during the production of the material. This method effectively solves the aforementioned shortcomings of external monitoring [15,16,17]. Sensors are mainly embedded in fiber stacks during FRP manufacturing; alternatively, some fibers can be removed from the FRP to create space for sensors. However, these methods may cause a concentration of stress at the interface between different materials due to the size of the sensor and resin accumulation caused by the internal cavity, leading to lower mechanical strength of the composite. Some scholars have proposed the concept of a self-sensing composite; by changing the material or adding other materials, mechanical strength can be maintained and sensing functions can be added.

In recent years, some researchers have proposed the concept of multifunctional composite materials [18], which, in addition to mechanical performance, combine other functional properties such as sensing performance, energy storage performance [19,20,21], etc. Multifunctional composite materials have a wide range of applications and are a trend for future development.

In terms of structural health monitoring, many scholars have proposed the concept of self-sensing composite materials [12,22,23,24,25]. Self-sensing composite materials are one type of multifunctional composite materials. By adding or replacing some of the materials in the fiber composite material with materials that have sensing capabilities, the composite material itself gains sensing capabilities. Different detection methods are used to quantify and locate material damage for timely maintenance and extended service life. Currently, self-sensing composite materials are mostly in the research and development stage and face many challenges.

The sensor technology currently used in the health monitoring of fiber composite materials can be mainly divided into external sensor technology, in which sensors are installed on the surface or outside of the material, and internal sensor technology, which can be further divided into embedded sensor technology and self-sensing composite materials. Self-sensing composites can be categorized as self-sensing fabrics and self-sensing matrices. Self-sensing fabrics are based on different sensing principles, including the use of the piezoresistive properties of carbon fibers to detect any strain on the material [12,22,23] as well as the use of piezoelectric fibers [26,27]. Conversely, the self-sensing matrix part is formed by adding a certain proportion of conductive material to the matrix of a polymer and utilizing the conductive material’s piezoresistive properties for sensing [28,29,30]; additionally, a piezoelectric polymer or conductive polymer can be employed as a matrix [31,32,33,34].

In recent years, PVDF seems to be a very promising material that can be used for energy harvesting and piezoelectric materials [35,36]. Generally, PVDF has four crystalline phases: α, *β*, γ, and δ phases [37]. In addition, there is another phase (ε phase) that can be obtained through a special process. The *β* phase is an all-trans conformation (TTTT); it is the crystal phase arrangement with the best polarity and piezoelectric properties. Therefore, much research has investigated how the overall *β*-phase content of the polymer can be increased [38]. The currently common methods are stretching, polarization, annealing heat treatment, and electrospinning [39].

Electrospinning is an electrohydrodynamic process [40], with its basic components consisting of a high-voltage power supply, injection pump, spinneret, and conductive collector. The process involves applying a high voltage electric field to a polymer solution, which is then emitted from the spinneret as a fluid jet. The fluid jet is stretched into a fiber by electrostatic forces, and the solvent evaporates during the stretching process, leaving a solid fiber that is collected on the grounded collector. This is one of the methods used to produce continuous microfibers and nanofibers. Nanofiber materials produced via this technique have a high surface area, high porosity, high aspect ratios, and other unique properties [41,42,43]. They have a wide range of applications in fields such as energy storage and healthcare. In sensor technology, for example, polyvinylidene fluoride (PVDF) can be electrospun to simplify the complex process of stretching and polarizing, resulting in microfibers and nanofibers with piezoelectric properties [26,44,45].

Because of the excellent characteristics of using electrospinning to produce PVDF fabric, in this study we propose a method combining electrospinning technology and FRP fabrication to obtain novel piezoelectric self-sensing composite laminates. This method produces a material that avoids the wiring difficulties encountered in previously proposed embedded-sensor technologies by taking advantage of the thickness of electrospun piezoelectric membranes and replacing glass fibers with carbon fibers to serve as electrodes. The proposed self-sensing composite laminate exhibits favorable mechanical performance and has high sensitivity to material damage.

## 2. Materials and Methods

### 2.1. Materials

For the electrospun PVDF composite membrane, pure PVDF powder was obtained (95.0 wt%, Alfa Aesar, Heysham, UK). Dimethyl sulfoxide (DMSO, 99.0 wt%) and acetone (99.5%) were obtained from Echo Chemical (Miaoli, Taiwan). Modified multiwalled carbon nanotubes (MWCNT-COOH, >95%), graphene nanoplatelets (GNPs, 1–5 nm), and dispersant were obtained from Conjutek (New Taipei, Taiwan).

For the self-sensing composite laminate, epoxy resin (2511-1A) and a hardener (2511-1BT) were obtained from Swancor Holding (Nantou, Taiwan) and mixed in a weight ratio of 10:3. Glass fibers (LT800) were obtained from Xptex Industries (Changhua, Taiwan). Carbon fibers (TAIRYFIL-TC-33 3K) were obtained from Formosa Plastic Group (Kaohsiung, Taiwan).

### 2.2. Preparation of Piezoelectric PVDF Composite Membrane

A piezoelectric PVDF composite membrane was fabricated using the electrospinning method (FES-COS Electro-spinning equipment, Falco, New Taipei, Taiwan). A PVDF (16 wt%) solution in DMSO and acetone (ratio 6/4 *w*/*w*) containing different concentrations (0.25–1.00 wt%) of MWCNT-COOH (i.e., CNTs) or graphene nanoplatelets (GNPs) and a given amount of dispersant was sonicated for 20 min, after which it was stirred for 12 h at 70 °C.

The solution was then placed in a plastic syringe with an iron needle with a 0.26 mm diameter tip (25G). Electrospinning was conducted at 15 kV at a feed speed of 2 mL/h. A cooking paper-covered rotating drum with a rotation speed (400 rpm) control function was used as the collector. The distance between the tip of the needle and the collector was 15 cm. The prepared PVDF composite membrane was collected on the cooking paper.

### 2.3. Characterization of Piezoelectric PVDF Composite Membrane

The morphology of the PVDF composite membrane obtained through electrospinning was examined using a high-magnification optical microscope (VHX-2000E, Keyence, Osaka, Japan). Attenuated total reflection Fourier-transform infrared (FTIR) (IRAffinity-1S, Shimadzu, Taiwan) spectra were obtained at room temperature and were used to evaluate the crystalline phase of the PVDF composite membrane.

The relative *β*-phase content was calculated using the following formula [39,46]:

(1) $$F(\beta )=\frac{A_{\beta }}{{(K}_{\beta }{/K}_{\alpha }{)A}_{\alpha }{+A}_{\beta }}$$

where A*_α_* and A*_β_* are the absorbance values at 766 and 840 cm^−1^, respectively, and K*_α_* and K*_β_* are the absorption coefficients of individual wavenumbers, which are 6.1 × 10^4^ and 7.7 × 10^4^ cm^2^/mol, respectively.

To determine the sensing performance of the PVDF composite membrane in practical applications, two types of composite membranes were preliminarily fabricated, with copper foil tape employed as sensor electrodes and PET plastic as outer packaging. The strain gauge was attached to the middle surface of the glass fiber composite material (GFRP), and a four-point bending test was performed to measure the piezoelectric signal under cyclic compression given fixed deformation conditions.

### 2.4. Fabrication of Self-Sensing Composite Laminate

**Figure 1:**
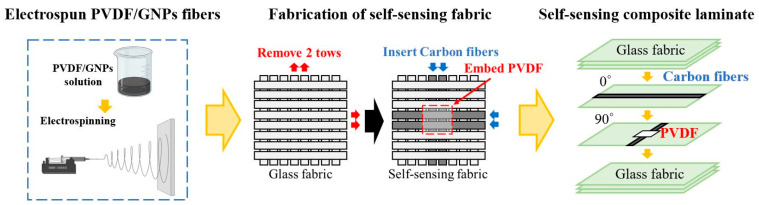
Schematic of fabricating the self-sensing composite laminate.

The self-sensing composite laminate developed in this study comprised eight layers of glass fiber fabric (Glass fiber LT800, Xptex Industries, Changhua, Taiwan) and a piezoelectric PVDF composite membrane interleaved at the laminate midplane. The stacking sequence was thus [0/90/0/90/PVDF/0/90/0/90]. The glass fibers located where the PVDF composite membrane was to be placed were replaced by carbon fibers, and the conductive properties of these fibers meant that the fibers could be used as sensor electrodes, conferring the laminate with both favorable mechanical and sensing properties. The self-sensing composite laminate was subjected to vacuum-assisted resin transfer molding. The ratio of epoxy resin to curing agent was 10:3. The epoxy was mixed with fiber cloth through vacuum infusion and then placed in an oven at 50 °C for 10 h to cure. For comparison, laminates with the same stacking sequence as the self-inductive laminate but lacking the PVDF composite membrane, with the PVDF composite membrane embedded in different positions and with differing ratios of carbon fiber replacement, were also fabricated. The specimens were cut into a rectangle of 100 mm × 25 mm × 2.5 mm. The self-sensing composite laminate fabrication process is illustrated in Figure 1.

Specimen description:

(1) Whether to replace glass fibers and embed a PVDF composite membrane.

**Figure 2:**
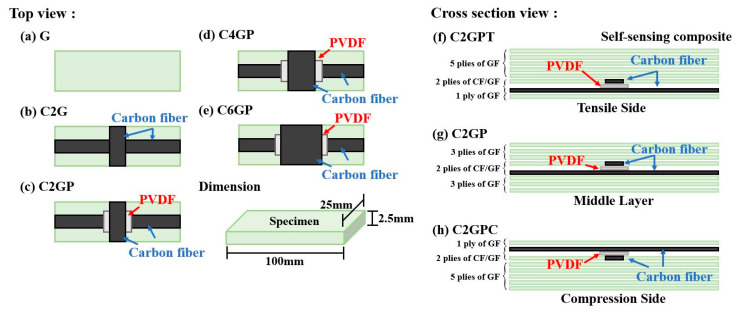
Schematic of different specimen types: (**a**) G; (**b**) C2G; (**c**) C2GP; (**d**) C4GP; (**e**) C6GP; (**f**) tensile-side membrane placement; (**g**) middle layer membrane placement; (**h**) compression-side membrane placement.

The effects of replacing glass fibers with carbon fibers and embedding an electrospun PVDF composite membrane on the mechanical properties of the GFRP were investigated. The notation G represents the pure GFRP specimen, C2G represents GFRP in which two perpendicular tows of glass fibers (G) were replaced with tows of carbon fibers (C), and P indicates that a PVDF composite membrane was embedded in the composite. Each test group consists of three specimens, with the specimen’s number located at the end of its code; for example, the three C2GP specimens are coded C2GP1, C2GP2, and C2GP3, respectively. A schematic of the different specimen types is shown in Figure 2a–c.

(2) Replacing different numbers of glass fibers

The effect of the number of glass fibers replaced with carbon fibers on the laminate’s mechanical properties was investigated. The notation C2G is the same as before, but there are also C4G and C6G, in which four and six lines of glass fibers were replaced, respectively. A schematic of the different specimen types is shown in Figure 2c–e.

(3) Different embedding positions

Different embedding positions represented the force type of the specimen in the four-point bending test. The C2G and P notations are the same as that for the other experiments; T represents the placement of the PVDF composite fiber film on the tension side, whereas C represents the placement on the pressure side. A schematic of the different specimen types is displayed in Figure 2f,g.

### 2.5. Four-Point Bending Test

**Figure 3:**
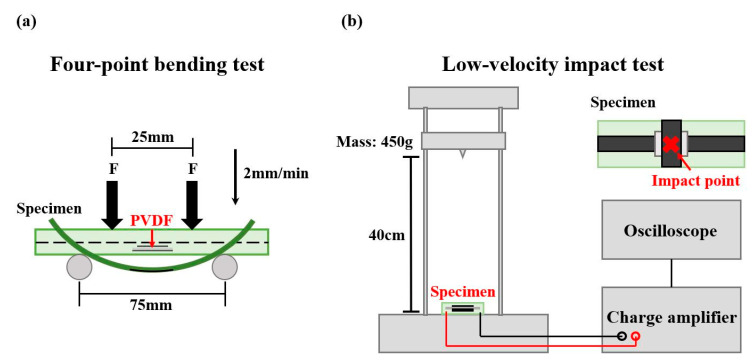
Experimental setup of the (**a**) four-point bending test and (**b**) low-velocity impact test.

The four-point bending test was performed using a universal testing machine (INSTRON 3365, Instron, Boston Metropolitan Area, MA, USA). The experimental setup is shown in Figure 3a. The lower span between the supports was 75 mm, and the pure bending zone in the middle had a width of 25 mm. A specimen was loaded to failure at a displacement rate of 2 mm/min. During the test, the specimen was connected to a charge amplifier (Piezo Film Lab Amplifier, TE Connectivity, Schaffhausen, Switzerland) with an electrode, and data were collected using the data acquisition system. The electrodes were welded to copper foil tape with wires and pasted onto the specimen with conductive silver paint. The experiment setup is illustrated in Figure 3a.

To calculate the flexural strength, the maximum bending stress of the material in the four-point bending process was estimated using classical beam theory; the formula is as follows [47]:

(2) $$\sigma =\frac{3Pa}{bh^{2}}$$

where *σ* is the bending stress, P is the total applied force measured by the load cell, *a* is the distance between the upper and lower spans, *b* is the width of the specimen, and *h* is the thickness of the specimen.

### 2.6. Low-Velocity Impact Test

The purpose of the low-velocity impact test was to investigate situations that may arise in practical applications, that is, when the self-sensing composite material is subjected to a strong enough impact to cause failure of its sensing ability. The results of the test would enable the selection of the embedded position in accordance with the requirements of practical applications.

PVDF composite membranes were embedded in the surface, middle, and bottom regions of each specimen to enable classification on the basis of the distance from the impact point. The low-velocity impact test was conducted using a self-developed low-velocity impact device. The specimen was fixed on the device and connected to a charge amplifier with copper wires to amplify the piezoelectric signal; an oscilloscope was used to record the peak value of the piezoelectric signal generated by the impact at various distances. The impact mass was 450 g, and the impact distance was between 1–40 cm.

According to the law of the conservation of energy, the potential energy of the impact mass was converted into kinetic energy as the mass fell (ignoring air resistance and friction), and when the mass hit the specimen, the energy was transferred to the specimen itself. Failure of sensing ability was determined on the basis of a change in the impact signal.

Gravitational potential energy formula:

(3) $$E_{p}(h)=mgh$$

where *E_p_* is the gravitational potential energy, *m* is the mass of the impact block, *g* is the gravitational acceleration constant (approximately 9.81 m/s^2^), and *h* is the height from which the mass block is released above the point of impact. Test groups were categorized on the basis of the embedded position away from the impact point, as shown in Figure 2f–h. In this test, S represents the surface of the specimen, M the middle, and B the bottom. Each type of specimen has 3 pieces numbered at the end of the code, for example, S-01. The experiment setup is illustrated in Figure 3b.

## 3. Results and Discussion

### 3.1. Characterization of PVDF Composite Membrane

To improve the piezoelectric properties of the electrospun PVDF fibers, carbon nanomaterial was added to the PVDF solution. MWCNT-COOH or GNPs were selected and added at 0.025–0.1 wt%.

**Figure 4:**
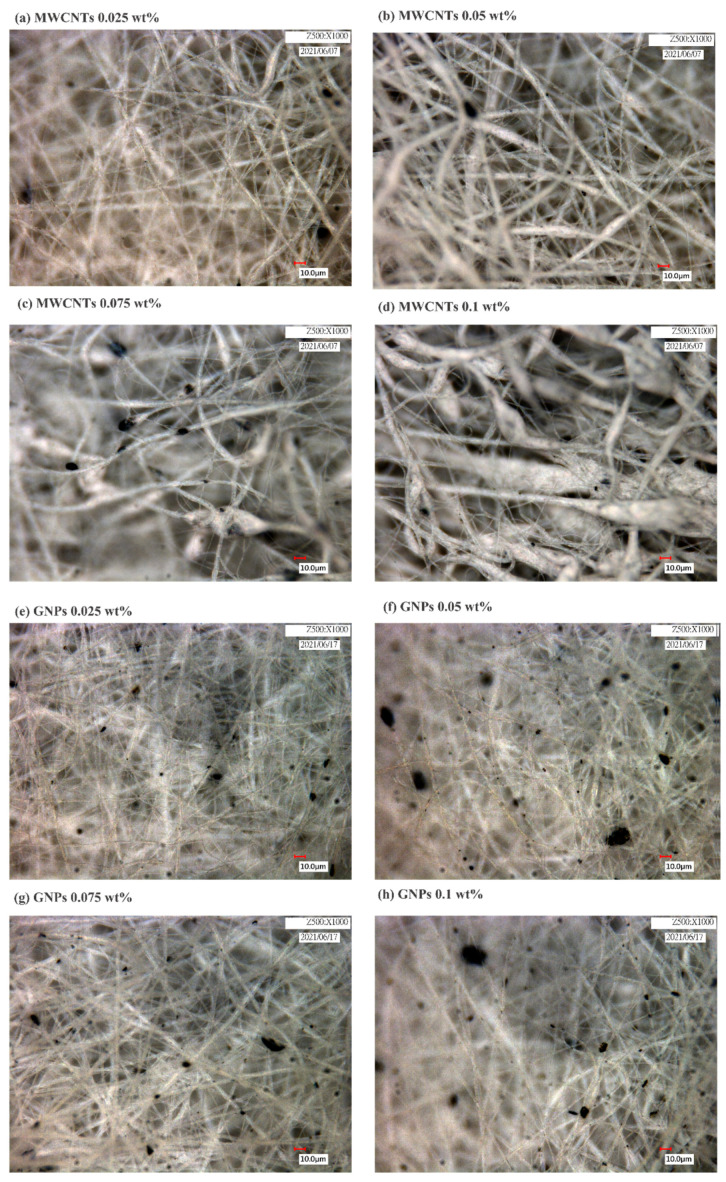
Optical microscope photographs demonstrating microstructural characterization of the electrospun polyvinylidene fluoride composite fibers with varying carbon nanotube content—(**a**) 0.025, (**b**) 0.05, (**c**) 0.075, and (**d**) 0.1 wt%—or graphene nanoplatelet content—(**e**) 0.025, (**f**) 0.05, (**g**) 0.075, or (**h**) 0.1 wt%.

According to the literature, carbon nanomaterials aggregate easily due to their aspect ratio, nanoscale, and the strong van der Waals forces between molecules; this aggregation means that they are difficult to disperse in a polymer matrix. One of the most effective ways currently to solve this problem is to chemically treat the carbon nanomaterial by adding functional groups, such as carboxyls (-COOH), to improve the nanomaterial’s dispersibility in a polymer matrix [48,49]. The aforementioned two carbon nanomaterials were thus selected and compared. Optical microscope photographs (Figure 4) show black spots in the fibers, which are aggregated CNTs (Figure 4a–d) or GNPs (Figure 4e–h). When the concentration of CNTs added was 0.075 wt% and 0.1 wt% (Figure 4c,d), the shape of the electrospun fibers was affected, and beaded fibers and clear aggregation of CNTs resulted. This was because the high CNT concentration led to the polymer solution having high viscosity. The aggregation of CNTs had a stronger impact on the properties of the polymer solution than did the aggregation of GNPs. Overall, the PVDF/GNP fibers were stable and less likely to aggregate into beads.

**Figure 5:**
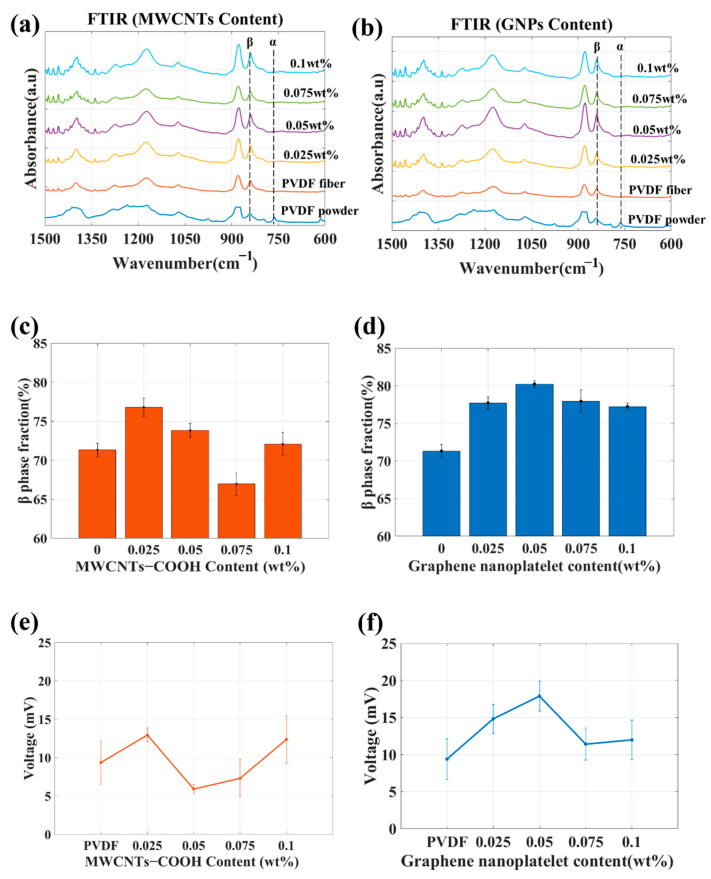
Fourier-transform infrared spectra of electrospun polyvinylidene fluoride composite fibers with (**a**) carbon nanotube or (**b**) graphene nanoplatelet content ranging from 0.0 to 0.1  wt%; relative *β*-phase content of polyvinylidene fluoride composite fibers with differing (**c**) carbon nanotube or (**d**) graphene nanoplatelet content; electrical output characteristics of electrospun PVDF composite fibers with differing (**e**) carbon nanotube or (**f**) graphene nanoplatelet content.

Whether adding CNTs or GNPs contributed to *β*-phase crystallization was investigated. The FTIR absorption spectra of the membranes containing CNTs or GNPs are displayed in Figure 5a and b, respectively; the relative *β*-phase content, calculated from these spectra, is depicted in Figure 5c,d. The PVDF composite fibers containing GNPs were superior to those containing CNTs in terms of fiber formation stability and relative *β*-phase content; the highest relative *β*-phase content was achieved with the addition of 0.05 wt% GNPs. The relative *β*-phase content of the fibers containing 0.05 wt% GNPs was approximately 9% higher than that of the pure PVDF fibers. These findings confirmed that GNPs enabled the reorganization of PVDF molecular chains during the electrospinning process in such a way that they crystallized on the surface, forming the *β* phase.

Regarding the response of the piezoelectric sensor, the peak trend in the piezoelectric signal was similar to that in the relative *β*-phase content, as illustrated in Figure 5e,f. The larger standard deviation of the piezoelectric signal for the PVDF/CNT-0.075 wt% and PVDF/CNT-0.1 wt% was related to fiber formation stability. Based on the presented results, the material with the highest performance was the PVDF/GNP-0.05 wt% composite fibers. Therefore, these composite fibers were embedded in the self-sensing composite laminate.

### 3.2. Mechanical Test of Self-Sensing Composite Laminate

**Figure 6:**
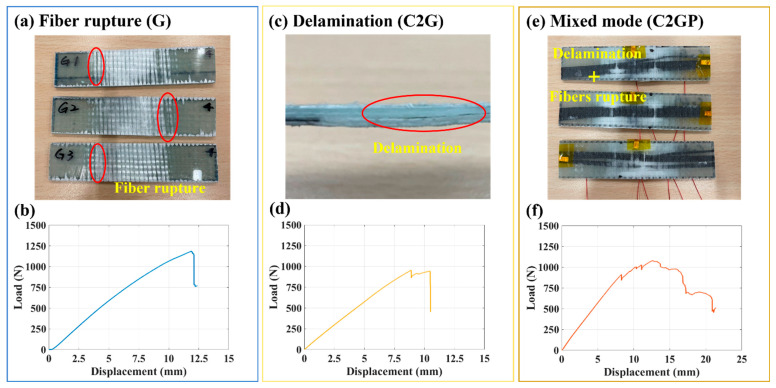
Photographs of specimens under different damage processes: (**a**) fiber rupture (G); (**b**) Load–displacement curves for (**a**); (**c**)delamination (C2G); (**d**) Load–displacement curves for (**c**); and (**e**) Mixed mode (C2GP); (**f**) Load–displacement curves for (**e**).

This experiment investigated the effects of replacing glass fibers with carbon fibers and embedding an electrospun PVDF composite membrane on the mechanical properties of GFRP. Figure 6 shows the load–displacement curves of the three specimens and the photographs of the failed specimens. The failure modes of the three types of specimens were different during the four-point bending test. Fiber rupture (Figure 6a) occurred instantly when the load limit of the GFRP specimen (G) was reached, and this rupture caused the specimen to fail, as shown in Figure 6b. The failure of the C2G specimen was due to delamination (Figure 6c) on the stack of the laminate containing carbon fibers; this delamination resulted in considerably lower mechanical strength than that of the G specimen (Figure 6d). The C2GP specimen failed due to the rupture of fibers (Figure 6e). Compared with the C2G specimen, the C2GP specimen had an extra PVDF membrane layer. Because of its high surface area and porosity, this membrane generated a bridging effect with the epoxy during the resin-mixing process, and this bridging effect enhanced interlayer adhesion and the laminate’s mechanical strength [50,51,52], thus reducing the likelihood of delamination in the specimen. Therefore, the trend was different from that for the C2G specimen (Figure 6f).

**Figure 7:**
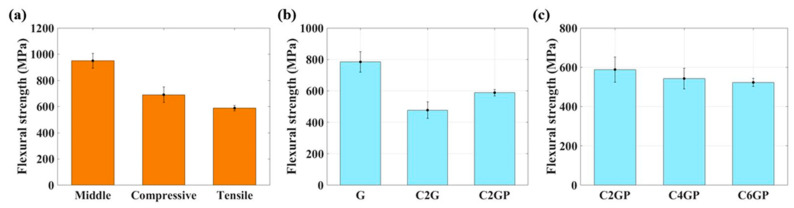
Flexural strength of (**a**) each specimen’s middle layer, compressive side, and tensile side; (**b**) G, C2G, and C2GP; and (**c**) C2GP, C4GP, and C6GP.

The results for the different embedding positions revealed that when the PVDF composite membrane was embedded on the tensile or compression side, the bending strength was lower than when it was embedded in the middle, as shown in Figure 7a. The influence of replacing different tows of glass fibers and embedding a PVDF composite membrane on the mechanical properties of the self-sensing composite was investigated. In the experiments, the PVDF composite membrane was embedded on the tension side. The results are presented in Figure 7b. The flexural strength of the C2G and C2GP specimens was lower than that of the pure GFRP possibly due to resin accumulation caused by the replacement of fiber materials. For C2G and C2GP, the embedded electrospun PVDF composite membrane increased the material’s flexural strength. The bridging effect was produced during the resin-mixing process, and this effect enhanced the adhesion between layers and improved the mechanical strength. Compared with C2G, the flexural strength of C2GP was 23.27% higher. The results also revealed that as the number of replaced glass fibers was increased, the flexural strength decreased slightly, as shown in Figure 7c. Compared with C2GP, the flexural strength of C4GP was approximately 7.79% lower, whereas that of C6GP was 11.13% lower. When the number of replaced glass fibers was higher, the degree of resin accumulation was also higher, and the arrangement of carbon fibers was difficult to control in a certain direction, thereby reducing the overall mechanical strength.

**Figure 8:**
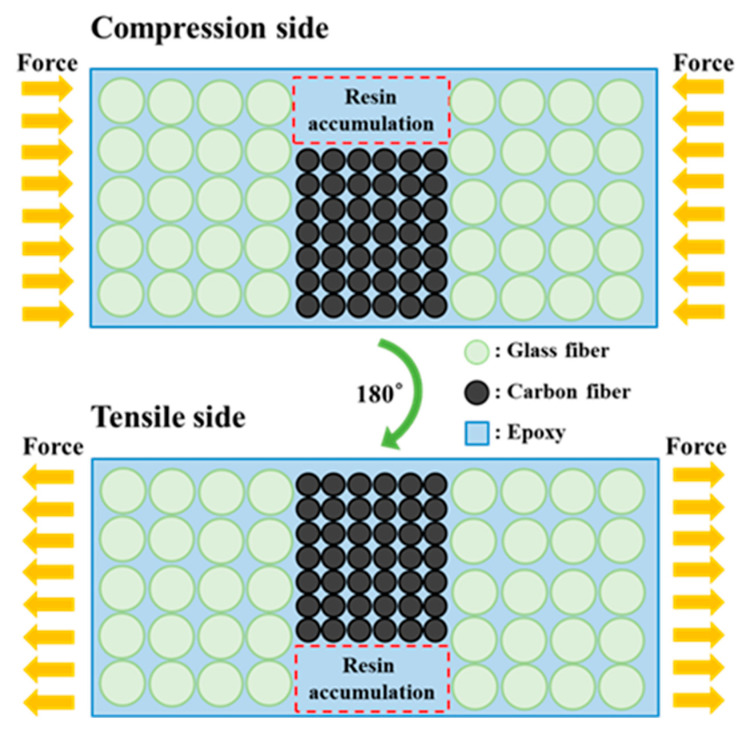
Schematic of resin accumulation inside the self-sensing composite laminate.

The reason that the bending strength when the PVDF composite membrane was embedded on the tensile or compression side was lower than that when it was embedded in the middle (see Figure 7a) was speculated to be due to the differing diameters of the carbon and glass fibers; the carbon fiber tows were not fixed in a weave, resulting in vacancies inside the material that were filled with resin. This caused the accumulation of resin in some locations. When stress was applied to the material, uneven stress existed at the material interface, as illustrated in Figure 8. The tensile strength of epoxy resin is generally lower than its compressive strength [53]. Thus, the bending strength of the specimen with a tensile-side-embedded membrane was low. The carbon fiber content of the entire composite laminate was very low. Although carbon fibers are stronger than glass fibers, their contribution to the laminate’s mechanical strength was small. For the specimen with a membrane embedded in the middle layer, the position of the membrane was close to the neutral axis; when the composite laminate was subjected to a bending moment, the glass fibers near the surface bore the force; thus, the bending strength was higher than that of the other two specimen types.

The flexural strength, damage modes, and damage locations of all specimens are listed in Table 1. The common damage modes of the composite laminates could be divided into matrix cracks, delamination, and fiber rupture (from mild to severe). During the four-point bending test, a specimen was gradually damaged in the area within the spans as the load was increased. The test instrument operation was discontinued when the bearing force monitored by the sensor decreased considerably (by ~40%) or the bearing force exceeded 4000 N. For most of the specimens, the fibers that provided most of the mechanical strength broke and stopped providing support before the test was terminated. Only the C2G specimens lost their support ability after a large visible area of delamination occurred between the layers of carbon fibers; thus, it can be speculated that the embedded electrospun PVDF composite fiber film enhanced the adhesiveness between layers, thereby improving the material’s mechanical strength, and the damage position can be speculated to have been mainly biased toward the membrane’s embedding position, partly due to the carbon fibers being replaced by hand rather than by machine. The carbon fiber replacement and weaving methods can be adjusted in the manufacturing process to reduce the degree of resin accumulation and thus solve this shortcoming.

### 3.3. Piezoelectric Response of Self-Sensing Composite Laminate

#### 3.3.1. Four-Point Bending Test

**Figure 9:**
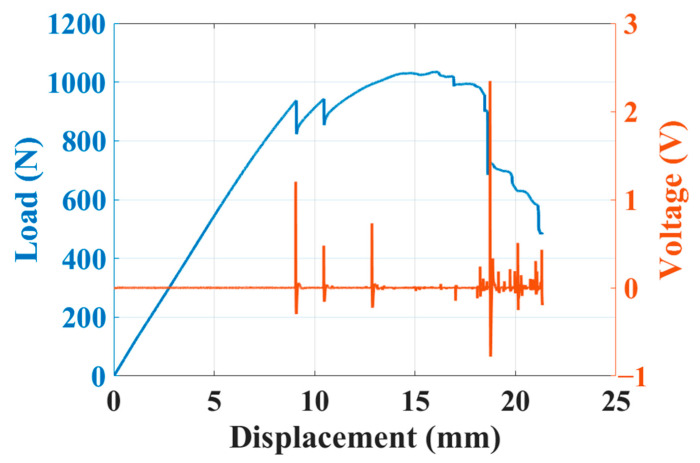
Load–displacement curve and piezoelectric response.

The results of the four-point bending test are presented in Figure 9. The blue curve is the load–displacement curve, whereas the orange curve denotes the piezoelectric response, which was measured synchronously. When the specimen was damaged during the bending test, such as when cracks appeared or delamination occurred, the internal piezoelectric PVDF composite membrane generated a corresponding piezoelectric response. When the load curve dropped for the first time, the specimen had begun to fail, and the strain energy that had accumulated during the bending process was released, resulting in a larger peak value of the piezoelectric response. When the specimen became damaged too severely, sensing performance failed. The main reasons for the failure were that the upper and lower carbon fiber electrodes short-circuited due to the damage, making normal measurement impossible, or the material damage caused the external copper foil electrodes to detach. According to the experimental results, the piezoelectric self-sensing composite laminate was confirmed to have sensing ability for preliminary damage monitoring.

#### 3.3.2. Low-Velocity Impact Test

The purpose of this experiment was mainly to investigate a more extreme situation that could arise during practical application—the self-inductive composite material being subjected to an impact that could lead to failure of its sensing ability. The results of this experiment allow for the selection of the optimal embedding position in accordance with the requirements of practical applications.

**Figure 10:**
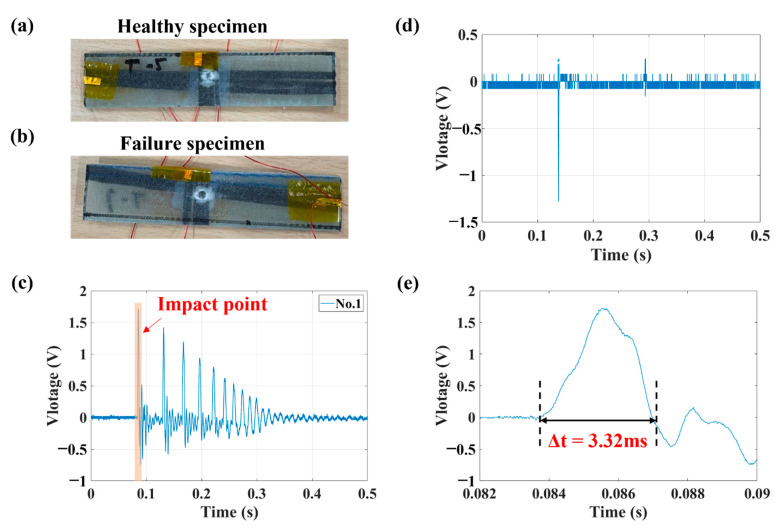
Photographs of a (**a**) healthy specimen and (**b**) failed specimen; piezoelectric response of a (**c**) healthy specimen and (**d**) failed specimen; (**e**) piezoelectric response (**a**) impact point.

According to photographs of the specimens (Figure 10a,b), because the degree of damage to the internal sensors of the specimens (and thus whether it has sensing ability) cannot be judged with the naked eye, it was judged on the basis of the change in the actual impact signal. The impact response of a healthy sensor specimen and a failed sensor specimen is shown in Figure 10c and d, respectively. When a similar damage signal to that shown in Figure 10d was observed, the sensing ability of the specimen was judged to have failed.

From the results presented earlier, we can obtain information about the impact on the self-sensing composite laminate material from the piezoelectric response. Here, we discuss the sensing capability of the membrane sensor at different depths of the composite laminate using three cases shown in Figure 2f, Figure 2g, and Figure 2h.

**Figure 11:**
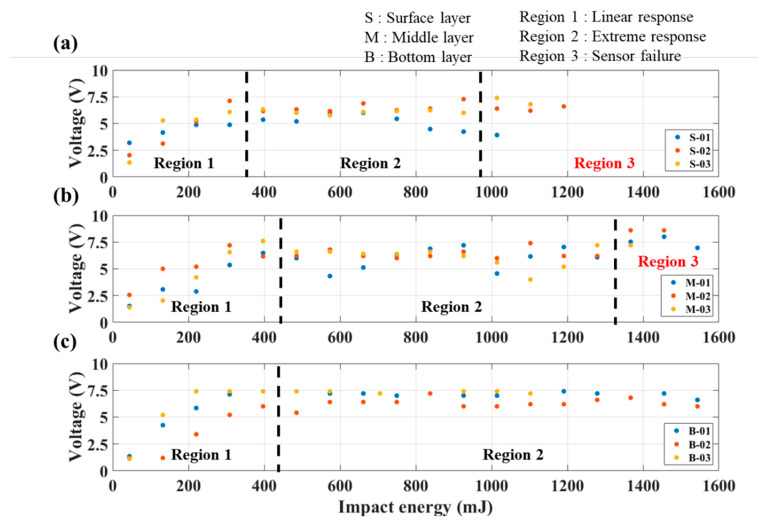
Piezoelectric response versus impact energy at the (**a**) surface, (**b**) middle, and (**c**) bottom of the specimen.

The results are presented in Figure 11, which is a plot of the first peak value of the impact response versus the impact energy. The plots can be divided into three main regions:

1. Linear response: The piezoelectric response in this region is positively linearly correlated with the impact energy; this corresponds to the region of normal applicability.
2. Extreme response: The piezoelectric response in this region is nonlinear due to the large impact energy; the response is thus the maximum peak value that the piezoelectric membrane can produce.
3. Sensor failure: In this region, due to the excessive impact energy, the composite laminate is severely damaged, and it has no sensing ability.

This experiment is conducted under extreme conditions (the impact point is directly above the sensing layer); therefore, the piezoelectric response is proportional to the material deformation within an allowable range. The piezoelectric response limit of the test specimen used in this experiment is approximately 7.5 V, and the deformation corresponding to different levels of impact energy will result in varying degrees of damage, which will affect the sensing performance of the test specimen.

The results revealed that before the impact energy reached approximately 400 mJ, the peak value of the piezoelectric response was positively linearly correlated with the impact energy. Once the impact energy exceeded 400 mJ, the peak value was approximately 6–8 V. The sensor failure region depended on the distance from the impact point. The surface of the specimen was closest to the impact point, and the material deformation caused by the impact was the most severe on the surface. The impact energy of the specimen exceeded 960 mJ, as shown in Figure 11a. The specimen’s measurement ability declined, and the damage could not be measured normally. The failure was speculated to have been caused by overlapping and the short-circuiting of the carbon fiber electrodes at the impact position due to material damage. The middle layer of the specimen began to exhibit damage when the impact energy exceeded 1300 mJ, as shown in Figure 11b. Because the bottom of the specimen was the farthest from the impact point, impact energy that was lower than 1600 mJ did not cause failure damage, as shown in Figure 11c.

From the results in Figure 11, the membrane sensor located near the surface (Figure 11a) is shown to be more fragile and prone to damage due to impact. The membrane sensor located deep in the structure (Figure 11c), by contrast, can maintain a good piezoelectric response, even when subjected to larger impact energy. In terms of application, different depths of the membrane sensor can be selected and adjusted according to different monitoring conditions.

## 4. Conclusions

In this study, electrospinning technology was used to fabricate a PVDF piezoelectric composite membrane that could serve as a sensing layer, and some fibers in glass fiber fabric were replaced with carbon fibers to solve the wiring difficulties often encountered in embedded sensor technology. A process for fabricating a self-sensing composite laminate was successfully established. This laminate has both favorable mechanical properties and sensing ability.

The influence of the position where the PVDF composite membrane was embedded on the mechanical properties of the laminate was investigated. The flexural strength was the lowest when the membrane was embedded on the tensile side, which was approximately 32.4% lower than that when the membrane was embedded in the middle. The flexural strength of the laminate containing an embedded PVDF composite fiber film was 23.27% (111 MPa) higher than that not containing such a film. With an increase in the number of carbon fiber tows that were replaced, the flexural strength decreased slightly.

Furthermore, at the practical application level, this study investigated the damage-sensing performance of self-sensing composite laminates through four-point bending and low-velocity impact tests. The results revealed that when damage occurred during bending, the piezoelectric response changed, confirming the preliminary sensing capability of the piezoelectric self-sensing composite laminate. The low-velocity impact experiment indicated the effect of impact energy on sensing performance and revealed the application and failure ranges of the self-sensing composite laminates. An impact energy level of 960 to 1300 mJ from the closest to furthest distance from the impact point led to the failure of the sensing ability of the self-sensing composite laminate.

This study sheds light on the future possibilities of advanced technology for composite SHM. A self-sensing composite can incorporate an artificially designed array of embedded membrane sensors in the composite structures. This proposed technique can be further strengthened by an improved fabrication process. The sensing module can potentially be coupled with the energy harvesting module, the structural energy storage module, and the wireless transmission module to enable real-time localized monitoring of the structure and achieve in situ self-sustaining SHM. Moreover, integrating the process of replacing fibers into the fiber-weaving production process is expected to significantly improve the mechanical performance and sensing capability of the self-sensing composites. All in all, the proposed concept has potential for large-scale production and commercialization.

## Figures and Tables

**Table 1:** Flexural strength, damage mode, and location of damaged specimens.

Specimen	Flexural Strength	Damage Mode	Damage Location
G1	709.398 MPa	F, D	C
G2	866.752 MPa	F, D	C
G3	774.419 MPa	F, D	C
C2G1	515.132 MPa	D	T
C2G2	403.293 MPa	D	T
C2G3	514.208 MPa	D	T
C2GP1	1009.445 MPa	F, D	T
C2GP2	872.840 MPa	F, D	T
C2GP3	968.182 MPa	F, D	C
C2GPC1	756.957 MPa	F, D	C
C2GPC2	614.208 MPa	F, D	C
C2GPC3	700.495 MPa	F, D	C
C2GPT1	616.146 MPa	F, D	C
C2GPT2	584.204 MPa	F, D	T
C2GPT3	565.659 MPa	F, D	T
C4GP1	513.814 MPa	F, D	T
C4GP2	571.842 MPa	F, D	T
C4GP3	542.341 MPa	F, D	T
C6GP1	554.551 MPa	F, D	C
C6GP2	502.585 MPa	F, D	T
C6GP3	510.966 MPa	F, D	T

F, fiber rupture; D, delamination; C, compressive side; and T, tensile side.

## Data Availability

The data presented in this study are available on request from the corresponding author.

## References

[1] Giurgiutiu V. SHM of aerospace composites–challenges and opportunities. Proceedings of the Composites and Advanced Materials Expo.

[2] Giurgiutiu V. (2020). Structural health monitoring (SHM) of aerospace composites. Polymer Composites in the Aerospace Industry.

[3] Ahmed O., Wang X., Tran M.-V., Ismadi M.-Z. (2021). Advancements in fiber-reinforced polymer composite materials damage detection methods: Towards achieving energy-efficient SHM systems. Compos. Part B Eng..

[4] Vishwanath R., Rohit B. A Review on Inspection and Maintenance of FRP Structures. Proceedings of the IOP Conference Series: Materials Science and Engineering.

[5] Gupta V., Sharma M., Thakur N. (2010). Optimization Criteria for Optimal Placement of Piezoelectric Sensors and Actuators on a Smart Structure: A Technical Review. J. Intell. Mater. Syst. Struct..

[6] LSwallow L.M., Luo J.K., Siores E., Patel I., Dodds D. (2008). A piezoelectric fibre composite based energy harvesting device for potential wearable applications. Smart Mater. Struct..

[7] Tuloup C., Harizi W., Aboura Z., Meyer Y., Khellil K., Lachat R. (2019). On the use of in-situ piezoelectric sensors for the manufacturing and structural health monitoring of polymer-matrix composites: A literature review. Compos. Struct..

[8] Ju M., Dou Z., Li J.-W., Qiu X., Shen B., Zhang D., Yao F.-Z., Gong W., Wang K. (2023). Piezoelectric Materials and Sensors for Structural Health Monitoring: Fundamental Aspects, Current Status, and Future Perspectives. Sensors.

[9] Wan X., Cong H., Jiang G., Liang X., Liu L., He H. (2023). A Review on PVDF Nanofibers in Textiles for Flexible Piezoelectric Sensors. ACS Appl. Nano Mater..

[10] Zhang M., Liu C., Li B., Shen Y., Wang H., Ji K., Mao X., Wei L., Sun R., Zhou F. (2023). Electrospun PVDF-based piezoelectric nanofibers: Materials, structures, and applications. Nanoscale Adv..

[11] Mohammadpourfazeli S., Arash S., Ansari A., Yang S., Mallick K., Bagherzadeh R. (2023). Future prospects and recent developments of polyvinylidene fluoride (PVDF) piezoelectric polymer; fabrication methods, structure, and electro-mechanical properties. RSC Adv..

[12] Abot J.L., Song Y., Vatsavaya M.S., Medikonda S., Kier Z., Jayasinghe C., Rooy N., Shanov V.N., Schulz M.J. (2010). Delamination detection with carbon nanotube thread in self-sensing composite materials. Compos. Sci. Technol..

[13] Ren B., Cho H., Lissenden C.J. (2017). A Guided Wave Sensor Enabling Simultaneous Wavenumber-Frequency Analysis for Both Lamb and Shear-Horizontal Waves. Sensors.

[14] Salmanpour M.S., Khodaei Z.S., Aliabadi M.H. (2016). Instantaneous Baseline Damage Localization Using Sensor Mapping. IEEE Sensors J..

[15] Bois C., Herzog P., Hochard C. (2006). Monitoring a delamination in a laminated composite beam using in-situ measurements and parametric identification. J. Sound Vib..

[16] Dziendzikowski M., Kurnyta A., Dragan K., Klysz S., Leski A. (2016). In situ Barely Visible Impact Damage detection and localization for composite structures using surface mounted and embedded PZT transducers: A comparative study. Mech. Syst. Signal Process..

[17] Ghoshal A., Chattopadhyay A., Schulz M.J., Thornburgh R., Waldron K. (2003). Experimental Investigation of Damage Detection in Composite Material Structures Using a Laser Vibrometer and Piezoelectric Actuators. J. Intell. Mater. Syst. Struct..

[18] Chung D.D.L. (2019). A review of multifunctional polymer-matrix structural composites. Compos. Part B Eng..

[19] Yu Y., Zhang B., Feng M., Qi G., Tian F., Feng Q., Yang J., Wang S. (2017). Multifunctional structural lithium ion batteries based on carbon fiber reinforced plastic composites. Compos. Sci. Technol..

[20] Shirshova N., Qian H., Shaffer M.S., Steinke J.H., Greenhalgh E.S., Curtis P.T., Kucernak A., Bismarck A. (2013). Structural Composite Supercapacitors. Composites Part A: Applied Science and Manufacturing.

[21] Chen H.-Y., Wu C.-Y., Hsueh Y.-T., Huang H.-H. (2021). Electromechanical properties of embedded multifunctional energy storage composite with activated carbon fiber/PVDF gel electrolyte. J. Chin. Inst. Eng..

[22] Nurprasetio I.P., Budiman B.A., Afwan A.A., Halimah P.N., Utami S.T., Aziz M. (2020). Nonlinear Piezoresistive Behavior of Plain-Woven Carbon Fiber Reinforced Polymer Composite Subjected to Tensile Loading. Appl. Sci..

[23] O’Donnell J., Chalivendra V. (2021). Multi-functional glass/carbon fibers hybrid inter/intra laminated composites. Compos. Part C Open Access.

[24] Chen X., Cheng S., Wang S., Wen K., Shi C., Zhang J., Zhao D., Han Y., Chen X., Li B. (2023). Embedding stretchable, mesh-structured piezoresistive sensor for in-situ damage detection of glass fiber-reinforced composite. Compos. Sci. Technol..

[25] Chen X., Cheng S., Wen K., Wang C., Zhang J., Zhang H., Ma H., Wu L., Li T., Li B. (2023). In-situ damage self-monitoring of fiber-reinforced composite by integrating self-powered ZnO nanowires decorated carbon fabric. Compos. Part B Eng..

[26] Lotfian S., Giraudmaillet C., Yoosefinejad A., Thakur V.K., Nezhad H.Y. (2018). Electrospun Piezoelectric Polymer Nanofiber Layers for Enabling in Situ Measurement in High-Performance Composite Laminates. ACS Omega.

[27] Hofmann P., Walch A., Dinkelmann A., Selvarayan S.K., Gresser G.T. (2018). Woven piezoelectric sensors as part of the textile reinforcement of fiber reinforced plastics. Compos. Part A: Appl. Sci. Manuf..

[28] Thostenson E.T., Chou T.-W. (2006). Carbon Nanotube Networks: Sensing of Distributed Strain and Damage for Life Prediction and Self Healing. Adv. Mater..

[29] Wang Y., Wang Y., Wan B., Han B., Cai G., Li Z. (2018). Properties and mechanisms of self-sensing carbon nanofibers/epoxy composites for structural health monitoring. Compos. Struct..

[30] Wang X., Chung D.D.L. (1995). Short-carbon-fiber-reinforced epoxy as a piezoresistive strain sensor. Smart Mater. Struct..

[31] Lynch J.P., Wang K.-W., Sohn H., Haghiashtiani G., Greminger M.A., Zhao P. (2014). Poling of PVDF matrix composites for integrated structural load sensing. Sensors and Smart Structures Technologies for Civil, Mechanical, and Aerospace Systems 2014.

[32] Greminger M., Haghiashtiani G. (2017). Multiscale modeling of PVDF matrix carbon fiber composites. Model. Simul. Mater. Sci. Eng..

[33] Haghiashtiani G., Greminger M.A. (2015). Fabrication, polarization, and characterization of PVDF matrix composites for integrated structural load sensing. Smart Mater. Struct..

[34] Das S., Yokozeki T. (2020). Polyaniline-based multifunctional glass fiber reinforced conductive composite for strain monitoring. Polym. Test..

[35] Černohorský P., Pisarenko T., Papež N., Sobola D., Ţălu Ş., Částková K., Kaštyl J., Macků R., Škarvada P., Sedlák P. (2021). Structure tuning and electrical properties of mixed PVDF and nylon nanofibers. Materials.

[36] Papež N., Pisarenko T., Ščasnovič E., Sobola D., Ţălu S., Dallaev R., Částková K., Sedlák P. (2022). A Brief Introduction and Current State of Polyvinylidene Fluoride as an Energy Harvester. Coatings.

[37] Kabir E., Khatun M., Nasrin L., Raihan M.J., Rahman M. (2017). Pure*β*-phase formation in polyvinylidene fluoride (PVDF)-carbon nanotube composites. J. Phys. D Appl. Phys..

[38] Pisarenko T., Papež N., Sobola D., Ţălu Ş., Částková K., Škarvada P., Macků R., Ščasnovič E., Kaštyl J. (2022). Comprehensive characterization of PVDF nanofibers at macro-and nanolevel. Polymers.

[39] Martins P., Lopes A.C., Lanceros-Mendez S. (2014). Electroactive phases of poly(vinylidene fluoride): Determination, processing and applications. Prog. Polym. Sci..

[40] He Z., Rault F., Lewandowski M., Mohsenzadeh E., Salaun F. (2021). Electrospun PVDF Nanofibers for Piezoelectric Applications: A Review of the Influence of Electrospinning Parameters on the beta Phase and Crystallinity Enhancement. Polymers.

[41] Jauhari J., Wiranata S., Rahma A., Nawawi Z., Sriyanti I. (2019). Polyvinylpyrrolidone/cellulose acetate nanofibers synthesized using electrospinning method and their characteristics. Mater. Res. Express.

[42] Jiang S., Chen Y., Duan G., Mei C., Greiner A., Agarwal S. (2018). Electrospun nanofiber reinforced composites: A review. Polym. Chem..

[43] Lee J.K.Y., Chen N., Peng S., Li L., Tian L., Thakor N., Ramakrishna S. (2018). Polymer-based composites by electrospinning: Preparation & functionalization with nanocarbons. Prog. Polym. Sci..

[44] Ali H.Q., Tabrizi I.E., Khan R.M.A., Zanjani J.S.M., Yilmaz C., Poudeh L.H., Yildiz M. (2019). Experimental study on dynamic behavior of woven carbon fabric laminates using in-house piezoelectric sensors. Smart Mater. Struct..

[45] Li Y., Liao C., Tjong S.C. (2019). Electrospun Polyvinylidene Fluoride-Based Fibrous Scaffolds with Piezoelectric Characteristics for Bone and Neural Tissue Engineering. Nanomaterials.

[46] Shao H., Fang J., Wang H., Lin T. (2015). Effect of electrospinning parameters and polymer concentrations on mechanical-to-electrical energy conversion of randomly-oriented electrospun poly(vinylidene fluoride) nanofiber mats. RSC Adv..

[47] Senokos E., Ou Y., Torres J.J., Sket F., González C., Marcilla R., Vilatela J.J. (2018). Energy storage in structural composites by introducing CNT fiber/polymer electrolyte interleaves. Sci. Rep..

[48] Cha J., Jin S., Shim J.H., Park C.S., Ryu H.J., Hong S.H. (2016). Functionalization of carbon nanotubes for fabrication of CNT/epoxy nanocomposites. Mater. Des..

[49] Punetha V.D., Rana S., Yoo H.J., Chaurasia A., McLeskey J.T., Ramasamy M.S., Sahoo N.G., Cho J.W. (2017). Functionalization of carbon nanomaterials for advanced polymer nanocomposites: A comparison study between CNT and graphene. Prog. Polym. Sci..

[50] Ekrem M., Avcı A. (2018). Effects of polyvinyl alcohol nanofiber mats on the adhesion strength and fracture toughness of epoxy adhesive joints. Compos. Part B Eng..

[51] Ekrem M. (2019). The effects of carbon nanotubes added polyvinyl alcohol nanofibers on mechanical properties of carbon reinforced composite laminates. Sādhanā.

[52] Saghafi H., Zucchelli A., Palazzetti R., Minak G. (2014). The effect of interleaved composite nanofibrous mats on delamination behavior of polymeric composite materials. Compos. Struct..

[53] Goglio L., Peroni L., Peroni M., Rossetto M. (2008). High strain-rate compression and tension behaviour of an epoxy bi-component adhesive. Int. J. Adhes. Adhes..

